# Novel Strategies in the Development of New Therapies, Drug Substances, and Drug Carriers Volume I

**DOI:** 10.3390/ijms23126635

**Published:** 2022-06-14

**Authors:** Andrzej Kutner, Geoffrey Brown, Enikö Kallay

**Affiliations:** 1 Department of Bioanalysis and Drug Analysis, Faculty of Pharmacy, Medical University of Warsaw, 1 Banacha, 02-097 Warsaw, Poland; 2 School of Biomedical Sciences, Institute of Clinical Sciences, College of Medical and Dental Sciences, University of Birmingham, Birmingham B15 2TT, UK; g.brown@bham.ac.uk; 3 Department of Pathophysiology and Allergy Research, Center of Pathophysiology, Infectiology & Immunology, Medical University of Vienna, Währinger Gürtel 18-20, A-1090 Vienna, Austria; enikoe.kallay@meduniwien.ac.at

At present, there is a strong need for new therapies that are effective and safe for widespread diseases. In developing new treatments, areas of current interest include drug development-related strategies, new therapeutic molecules, and new drug delivery systems. The Special Issue was conceived to promote synergy between research and industrial activities in the design and development of new drugs and, therefore, was not limited to any specific aspect of development. It covers the entire process from the identification of a molecular target, studies of drug substance–protein interactions, the modeling and optimization of the functional activity, design and chemical synthesis, biological evaluation, and the development of new pharmaceutical carriers.

The original articles and reviews are focused on the design and development of new anticancer treatments, new anticancer low-molecular-weight agents as potential drug substances, and the elucidation of their mechanisms of action. The Issue also includes studies on novel modulators of the serotonergic system used to treat central nervous system disorders, novel agents against infectious diseases, and the development of anti-plasmodial and anti-inflammatory agents. The successful identification of new compounds for development as a drug substance is coming from the rich source of medicinal plants and medicinal chemistry approaches. The molecular mechanism of potential drug substances is important to their development as a drug.

The emergence of drug-resistant parasites makes malaria one of the most dangerous infectious diseases. In their research to fight this disease, Nawrot et al. [1] have examined a combination of macromolecular and low-molecular-weight compounds obtained from the latex-bearing plants, including *Chelidonium majus* L. This is a medicinal latex-bearing plant that has been used in traditional folk medicine to treat human papillomavirus (HPV)-caused warts, papillae, and condylomas. The authors have identified the novel major latex protein CmMLP1 and presented a model of its structure. CmMLP1 and the accompanying three alkaloids decreased the in vitro viability of human cervical cancer cells (HPV-negative and HPV-positive).

Gracz-Bernaciak et al. [2] have reviewed the general role of latex, a key part of a plant’s defense system, in plant physiology. The authors have described a broad spectrum of active components that are beneficial not only for plants but also for human health. Examples include morphine and codeine from poppy latex. The significance of alkaloids and proteins to the defense system of plants was analyzed for *Chelidonium majus* L, from the poppy family. Their investigations of medicinal latex compounds are outlined, including functional studies of proteins and other pharmacological compounds, via the use of techniques such as CRISPR/Cas9 gene editing.

The pharmacological actions of isoflavones of natural origin, such as genistein (GE), relate to their antioxidant activity, and they protect cells from carcinogenesis. Using a self-assembled monolayer on the gold electrode in simulated voltammetry, Stolarczyk et al. [3] have revealed that a new thiolated genistein analog (TGE) has antioxidant activity. The electroactive centers of TGE and its oxidation mechanism have been elucidated using infrared spectrometry supported by quantum chemical and molecular mechanics calculations. In vitro studies indicated that TGE exhibits a high cytotoxic activity towards the human prostate cancer cell line DU145 and that its activity against normal prostate epithelial cells is lower than that of GE.

Zagórska-Dziok et al. [4] studied the kinetic release of genistein (GE) from biomaterials such as polymeric hydrogels and observed favorable physicochemical properties and biocompatibility of the active substance. Non-toxic poly(chitosan-ester-ether-urethane) hydrogels were synthesized by ring-opening polymerization (ROP) and polyaddition. The release rate of GE from hydrogels was near-zero-order kinetics without “burst release” and with non-Fickian transport. The non-toxicity of hydrogels, together with a relatively highly controlled release profile of GEN, suggests that polymeric hydrogels are an effective drug substance carrier.

Drug substances of a natural origin continue to provide drug candidates that are effective against a variety of diseases. However, medicinal chemistry represents a dominant approach to new drug candidates and is well represented by papers in the Special Issue.

By using an advanced medicinal chemistry approach, Jaromin et al. [5] have identified a very high anti-plasmodial activity and selectivity, and a lack of cytotoxicity, for a diastereomeric mixture of *N*-(3,3-dimethylbutyl)-1-octyl-2,3,4,9-tetrahydro-1H-pyrido [3,4-b]-indole-3-carboxamides. The racemic mixture decreased the in vitro viability of human cervical cancer cells, including both HPV-negative and HPV-positive. In silico simulations have revealed possible interactions of the mixture with the enzymes that are essential for parasite metabolism.

Ovarian cancer is one of the most lethal cancers in women, and the current therapies are not sufficiently effective. Piątek et al. [6] have studied a potential new treatment strategy regarding the effectiveness of vitamin D against ovarian cancer cell lines. Synthetic analogs of the active hormonal form of vitamin D have different potencies against high-grade serous ovarian cancer cell lines. The efficacy of the most active analogs in increasing *CYP24A1* expression was cell line- and chemical-structure-dependent. Therefore, optimizing the chemical structure of analogs together with screening for activity in ovarian cancer cells might offer the prospect of a new treatment for ovarian cancer.

Vitamins D and plant polyphenols are both therapeutic anticancer agents and chemo-preventive. Their mechanism of synergistic action is not fully elucidated. Maj et al. [7] have evaluated the biological effects of an active vitamin D metabolite and the plant polyphenol resveratrol (RESV) on lung cancer cells. The effectiveness of both agents was dependent on the genetic nature of the cells. The effect of vitamin D metabolite on the induction of *CYP24A1* expression was enhanced by RESV. There was a pronounced effect of the compounds on cytokine production. The authors have postulated that the differences in the responsiveness of the cell lines to vitamin D metabolite and RESV relates to inherent epigenetic differences.

Recent studies of transcription have revealed the principles that govern the action of vitamin D action on a genome-wide scale. Even so, the identification of analogs that are potentially useful therapeutically has had limited success. Pike and Meyer [8] have postulated that a better understanding of the action of vitamin D at the transcriptional level, by the identification of sequential molecular events that occur during activation of most genes, both in vivo and in vitro studies, may provide insight into beneficial structure/activity relationships. Therefore, a novel approach to identifying potent anticancer vitamin D analogs would be to identify the extent to which they lead to selective gene expression within certain cells and tissues.

To facilitate the selection of vitamin D analogs as potential drug candidates, Yasuda et al. [9] have developed and reviewed in vitro systems, examining their affinity for VDR and CYP24A1-mediated metabolism. The authors have developed an in vivo system, including a *Cyp27b1* gene-deficient rat (a type I rickets model), a *Vdr* gene-deficient rat (a type II rickets model), and a rat with a mutant *Vdr* (R270L) (another type II rickets model), by using genome editing. These models can be used to determine the efficacy of vitamin D analogs, the readout being the amelioration of the symptoms of rickets.

Fluorine substitution of drug substances offers a number of advantages, including changing the pKa and dipole moment of the molecule, improving the chemical or metabolic stability, and enhancing the binding affinity to the target protein. These unique features of fluorinated compounds encouraged Kittaka et al. [10] to review the molecular structures of fluorinated analogs of vitamin D, their synthetic methodologies, and the resulting biological activities, including their anticancer action. Fluorination of the CYP24A1 metabolic positions at the side chain of vitamin D led to strong VDR agonists that have a prolonged half-life in vivo.

Chrzanowska et al. [11] have designed and synthesized copper (II) complexes with 3-(4-chloro-3-nitrophenyl)thiourea. They were cytotoxic against human cancer cell lines at the low micromolar range, and this concentration did not affect normal cells. The complexes also induced lactate dehydrogenase (LDH) release from the cancer cell lines. They provoked the immediate apoptosis of cancer cells. The complexes also reduced the level of interleukin-6. An effect of the complexes on the detoxifying and reactive oxygen species of cells that scavenge tumor cells was demonstrated.

The use of methotrexate (MTX) chemotherapy is hampered by a lack of selectively against the target tumor. To improve this therapy, Woźniak et al. [12] have designed and evaluated a novel glucose–methotrexate conjugate (GLU–MTX), whereby a cleavable linkage allows the intracellular release of MTX after selective uptake through the glucose transporter−1 (GLUT1). GLU–MTX inhibited the growth of colorectal, breast, and lung adenocarcinomas, squamous cell carcinoma, and osteosarcoma cell lines. For these cells, GLU–MTX uptake was increased 17-fold as compared with unconjugated MTX. In a mouse model of breast cancer, the GLUT–MTX conjugate caused growth inhibition in vivo.

Currently, there are limited treatments for metastatic osteosarcoma (OS). However, alpha-ketoglutarate (AKG) may represent a novel adjuvant therapy, and Kaławaj et al. [13] have tested whether supplementation of osteosarcoma cell lines with exogenous AKG exerts an anti-cancer effect. AKG inhibited the proliferation of the OS cell lines in a concentration-dependent manner. AKG blocked cell cycle progression at the G_1_ stage, which was accompanied by a decrease in the level of cyclin D1. AKG activated both initiator and executioner caspases and induced the expression of Bax while inhibiting Bcl2.

Colorectal cancer (CRC) is the third most frequently diagnosed cancer in men and the second in women. The effectiveness of standard cisplatin therapy for CRC is low due to its lack of specificity and the frequent development of drug resistance. There are also severe side effects. Gurba et al. [14] have documented that Au(III) complexes are promising drug candidates for CRC treatment due to their structural similarity to Pt(II). The authors have reviewed the efforts that have led to stable Au(III) complexes that have potentially selective cytotoxic activity for cancer cells. Unexpectedly, rather than binding to DNA, the inhibition of proteins, such as thioredoxin reductase, mediates the anticancer effects of the complexes.

In the search for safer and more effective anti-inflammatory agents, Szczukowski et al. [15] have designed and synthesized a broad series of novel *N*-substituted-1,2,4-triazole-based derivatives of pyrrolopyridazinone. The derivatives showed significant inhibition of the activity of cyclooxygenase-2 (COX-2) and promising COX-2/COX-1 selectivity. A molecular docking study has demonstrated that the new derivatives occupied, in the active site of COX-2, a position that usually binds meloxicam, a known NSAID. The new derivatives increased the viability of cells that were pre-incubated with the pro-inflammatory lipopolysaccharide and reduced the level of reactive oxygen and nitrogen species (RONS) during induced oxidative stress.

Disturbances in serotonergic neurotransmission are closely related to central nervous system disorders such as depression, anxiety, and schizophrenia, and Król et al. [16] have searched for novel modulators of the serotonergic system. The authors have designed and synthesized novel 4-aryl-pyridopyrimidines. The compounds demonstrated very high binding affinities for the 5-HT_1A_ receptor and high affinities for the D_2_, 5-HT_2A_, and 5-HT_7_ receptors. The lead compound had the activity profile of a presynaptic agonist.

The complex nature of the serotonergic system and interactions with other neurochemical systems indicate that the development of depression may be mediated by various pathologic mechanisms, including disturbance to the transmission within the central 5-HT synapses. Ślifirski et al. [17] have reviewed the potential that the serotonergic system offers for the development of new antidepressant therapies. Of importance is a combination of serotonin inhibition together with different agents that are directed towards the 5-HT system. In this regard, the authors summarized recent searches for new antidepressants.

A substantial subpopulation of depression patients is unresponsive to current therapies, and so there is a need for therapies that are more effective and also more tolerable. Pharmacological regulation of histone acetylation levels is considered a potential clinical strategy. Histone acetylation status is also a potential diagnostic biomarker for depression. Inhibitors of histone deacetylases (HDACs) are widely studied as novel therapeutics. Park et al. [18] have reviewed the histone acetylation status in depression and the therapeutic potential of HDAC inhibitors.

Prostate cancer is the most common cancer in men. For patients with advanced and metastatic disease, the available treatment is limited to delaying the progression of the tumor. Couty et al. [19] have examined a natural multifunctional antimicrobial peptide, dermaseptin-B2, that has shown some antitumor activity. To improve its pharmacological properties and decrease its peripheral toxicity and lethality, the authors have developed a hormonotoxin molecule composed of dermaseptin-B2 combined with d-Lys^6^-LHRH protein to target the luteinizing hormone-releasing hormone (LHRH) receptor. The new hormonotoxin reduced tumor burden in mice and was better tolerated than dermaseptin-B2, as it induced cell death by apoptosis rather than necrosis.

Calcimimetics, the pharmacological allosteric agonists of the extracellular calcium-sensing receptor (CaSR), show substantial gastrointestinal side effects and induce the expression of inflammatory markers (e.g., IL-8) in colon cancer cells. Schepelman et al. [20] have used both CaSR-specific (*R*) and -unspecific (*S*) enantiomers of a calcimimetic (NPS 568) and a calcilytic (allosteric CaSR antagonist; NPS 2143) to prove that these effects are mediated via the CaSR. As expected, only the CaSR-selective *R*-enantiomer of the calcimimetic induced CaSR and IL-8 expression, an effect inhibited only by *R*-NPS-2143 but not *S*-NPS-2143. The investigators have, therefore, proved that the pro-inflammatory effects of calcimimetics in colon cancer cells are mediated through CaSR activation.

In searching for an efficient anticancer molecule that targets the human DNA topoisomerase IB, Soren et al. have [21] investigated the catalytic steps of human DNA topoisomerase IB in the presence of a drug substance coded MMV024937, which was obtained from the open-access drug bank Medicines for Malaria Venture. The substance strongly and irreversibly inhibited the cleavage activity of the enzyme and reduced the cell viability of cancer cell lines. Molecular docking and molecular dynamics simulations have suggested that MMV024937 binds to the human DNA topoisomerase IB-DNA complex and sits inside the catalytic site of the enzyme. The investigators have, therefore, provided a molecular-level explanation for the cleavage-inhibition effect of the substance and a possible rationale for using this drug as a lead for the development of anticancer agents.

In summary, the papers within the Special Issue have cover a wide aspect to the development of new drug substances. Medicinal chemistry, supported by molecular modeling and quantum mechanical calculations, is at present the leading approach to drug discovery. The exploration of plant biodiversity is still an important avenue in this field.
